# Infectious outcomes of a standardized subcutaneous immunoglobulin dose reduction strategy in primary immune deficiencies amid global shortage

**DOI:** 10.3389/fimmu.2024.1527514

**Published:** 2025-01-20

**Authors:** Pedro Moral Moral, Marta Dafne Cabanero-Navalon, Paula Teresa López-León, Héctor Balastegui-Martín, Sandra Martínez Mercader, Amparo Mir, Victor Garcia-Bustos

**Affiliations:** ^1^ Primary Immunodeficiencies Unit, Department of Internal Medicine, University and Polytechnic Hospital La Fe, Valencia, Spain; ^2^ Research Group of Chronic Diseases and HIV Infection, Health Research Institute La Fe, Valencia, Spain; ^3^ Central Research Unit, University of Valencia, Valencia, Spain; ^4^ Severe Infection Research Group, Health Research Institute La Fe, Valencia, Spain; ^5^ Unit of Infectious Diseases, University and Polytechnic Hospital La Fe, Valencia, Spain

**Keywords:** immunoglobulin replacement therapy, humoral primary immune deficiencies, subcutaneous immunoglobulin, infections, cost-effectiveness, resource shortage

## Abstract

**Introduction:**

Immunoglobulin replacement therapy (IgRT), either intravenous (IVIg) or subcutaneous (SCIg), is crucial for managing primary immune deficiencies (PIDs) with hypogammaglobulinemia by reducing infection rates and mortality. During the COVID-19 pandemic, a global shortage of SCIg prompted our unit to reduce SCIg doses or maintain the same dose intravenously. This study evaluates the impact of a standardized SCIg dose reduction on infection rates and clinical outcomes in patients with humoral PID and with a low burden of infections.

**Methods:**

Adult PID patients on SCIg for at least 6 months, with IgG trough levels ≥ 700 mg/dL (or ≥ 900 mg/dL under specific conditions), and no significant infections in the past 6 months were eligible. A dose reduction of 15 mg/kg/week (60 mg/kg/month) for every 150 mg/dL above 700 mg/dL (or 900 mg/dL) was proposed. Clinical and laboratory data, and infectious events at 6- and 12-month follow-ups, were analyzed.

**Results:**

Thirty-one patients with PID were included: common variable immunodeficiency (54.83%), IgG subclass deficiency (9.67%), and other PIDs (35.48%). The average SCIg dose was initially reduced from 7.82 g/week to 5.72 g/week and adjusted to 6.94 g/week at 12 months. There was no significant change in severe or mild infections before and at 6- and 12-months post-dose adjustment. The dose reduction saved an average of 5,550 euros per patient annually, totaling 172,050 euros annually for our cohort.

**Discussion:**

Optimizing SCIg doses in selected well-controlled humoral PIDs is feasible without increasing infection rates, conserving this plasma-derived product during shortages. Larger prospective studies are needed to confirm this strategy's utility and its application to other Ig formulations.

## 1 Introduction

Primary immunodeficiencies (PID) represent a heterogeneous group of diseases characterized by abnormalities in one or more components of the immune system. Humoral immunodeficiencies are the most frequent PID, constituting around 30-70% of the total PIDs with specific defects involving a dysfunction or absence of B cell lymphocytes, with a consequently decrease in the production of immunoglobulins (Ig) that recognize specific antigens, facilitating their elimination (1) These patients mainly suffer from severe or recurrent infections (2), and some may suffer autoimmune and neoplastic complications due to immune dysregulation (3) which constitute, nowadays, its main cause of morbidity and mortality (4, 5).

Immunoglobulin replacement therapy (IgRT), administered either intravenously (IVIg) or subcutaneously (SCIg), is essential in managing humoral PIDs. It has been shown to reduce infection frequency and severity, decrease organ damage rates, and lower patient mortality (6, 7). Both IVIg and SCIg have demonstrated similar efficacy levels, although SCIg is associated with fewer systemic adverse events, improved quality of life, and reduced costs (8, 9).

The correlation between IgG trough levels and reduced infection rates is well stablished (10–12). Current clinical guidelines recommend an initial IgRT dose of 400-600 mg/kg/month to achieve steady-state trough IgG levels of 600-800 mg/dL (11, 13–15). However, there are no defined protocols to adjust subsequent dosing and defining the ideal IgRT dose for each patient remains a challenge, as individual biological thresholds vary (11, 16–18). In this regard, patients with bronchiectasis, interstitial lung disease (ILD), autoimmune cytopenias, and enteropathy, may require higher IgG trough levels (11, 16). This necessitates careful dose optimization to avoid undertreatment, which increases infection risk, or overtreatment, especially regarding increasingly scarce availability of plasma-derived products, specifically Igs.

Taking advantage of the global shortage of Ig during the COVID-19 pandemic, the objective of this study was to retrospectively evaluate the impact of a standardized reduction in SCIg doses on infection rates and clinical outcomes in patients with humoral PID who have maintained stable high IgG trough levels and have not experienced serious or recurrent infections. We aim to provide evidence for individualized dosing strategies to prevent potential IgRT overdosing in humoral PID patients, while addressing the challenges associated with the limited availability of plasma-derived products.

## 2 Material and methods

### 2.1 Study design and inclusion criteria

**Figure 1:**
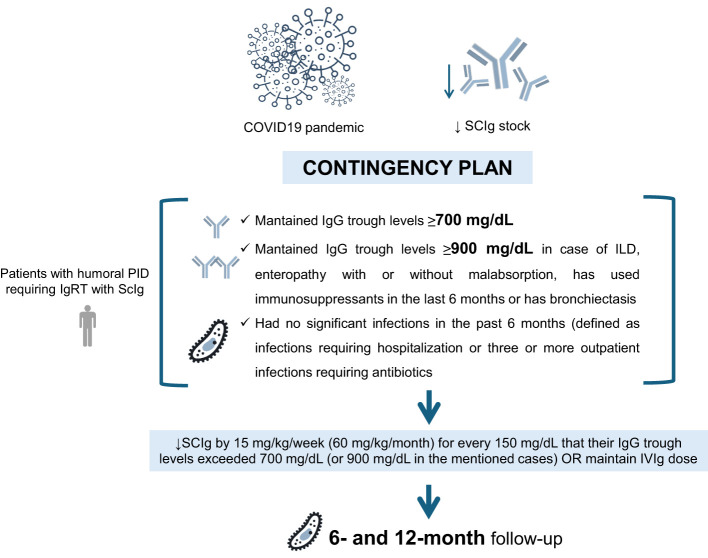
Contingency plan for subcutaneous immunoglobulin (SCIg) dose adjustment in humoral primary immunodeficiency (PID) patients during the COVID-19 pandemic in the Unit for Primary Immunodeficiencies from the University and Polytechnic Hospital La Fe. IgRT, immunoglobulin replacement therapy; IVIg, intravenous immunoglobulin.

In 2021, coinciding with a severe global shortage of Ig due to the COVID-19 pandemic, hospitals across Spain experienced a drastic reduction in their stock of SCIg. To address this exceptional situation, the Unit for Primary Immunodeficiencies from the University and Polytechnic Hospital La Fe, implemented a contingency plan from a clinical perspective, which consisted of (1) a thorough revision of the clinical indications for IgRT for each patient, and (2) a dose adjustment in the dose of SCIg in patients with humoral PID who had stable clinical and IgG trough levels. Thus, patients over 18 years who were receiving SCIg for at least 6 months, maintained IgG trough levels of 700 mg/dL or higher (or more than 900 mg/dL in those patients who had ILD, enteropathy with or without malabsorption, use of immunosuppressants in the last 6 months or presence of bronchiectasis), and had no significant infections in the past 6 months (defined as infections requiring hospitalization or three or more outpatient mild infections requiring antibiotics) were eligible for dose adjustment. All patients who met the dose adjustment criteria were given the option to either reduce their SCIg dose as previously mentioned or switch to IVIg at the same dose they were receiving subcutaneously (**Figure 1**).

All patients were evaluated for these criteria in May 2021. Those who were eligible were proposed to reduce their SCIg regimen by 15 mg/kg/week (60 mg/kg/month) for every 150 mg/dL that their IgG trough levels exceeded 700 mg/dL (or 900 mg/dL in the mentioned cases) (**Figure 1**). For example, a 70 kg patient showing IgG trough levels of 1000 mg/dL and without relevant infections in the past 6 months, would have their dose reduced by 2 g per week or 8 g per month. One year after completing this clinical contingency plan, data were retrospectively collected to conduct the scientific study.

### 2.2 Data collection and variables

Clinical data were obtained retrospectively from electronic medical records and recorded in a database. The demographic characteristics of each patient were documented, encompassing age, sex, age at onset of immunodeficiency symptoms, age at diagnosis, and specific PID diagnosis. The presence and type of infections prior to dose adjustment were recorded, categorizing them into major bacterial infections (pneumonia, meningitis, osteomyelitis, intra-abdominal infections, cellulitis or soft tissue infections, sepsis, or opportunistic infections) and mild infections (upper respiratory tract infections, lower respiratory tract infections, gastrointestinal infections, urinary tract infections, and soft tissue infections). Additionally, the average number of emergency visits and hospitalizations per year was documented. Non-infectious comorbidities including autoimmune cytopenias, lymphadenopathies, splenomegaly, hepatomegaly, and clinical or imaging findings of portal hypertension, systemic autoimmune disorders, lung, gastrointestinal, cutaneous, and neurological involvement, as well as malignancy (both solid and hematological neoplasia) were documented. The infectious processes that the patient suffered at 6 and 12 months after the dose adjustment were documented, including bacterial major and mild infections.

Laboratory variables including IgG trough levels (mg/dL), IgM (mg/dL), IgA (mg/dL), total number of leukocytes (cell/μL), neutrophils (cell/μL), CD3 cell count (cell/μL), CD4 cell count (cell/μL), CD8 cell count (cell/μL), CD4/CD8 ratio, CD19 cell count (cell/μL), and natural killers (NK) cell count (cell/μL) prior to dose adjustment, and at 6 and 12 months of follow-up were recorded. Regarding the treatment, the type of SCIg preparation (conventional SCIg 20% or hyaluronidase-facilitated SCIg 10%) and their brand, the SCIg dosage the patient received prior to adjustment, and the subsequent dosage after 6 and 12 months of follow-up were documented, as well as the immunosuppressant received therapy, if any.

### 2.3 Statistical analysis

The statistical analysis was conducted using the statistical software R, version 4.0.1. Continuous variables were described by their mean and standard deviation. Discrete variables were presented through the distribution of frequencies and percentages.

Normality was assessed by quantile-quantile QQ plots and the Shapiro-Wilk test. Comparisons between the parameters prior to the dose adjustment and after a 6- and 12-month follow-up period were made using the Cochran Q test for categorical variables and the Friedman test for quantitative variables. Statistical significance was defined as p < 0.05. A generalized linear mixed model was developed to evaluate the effect of different time points on the frequency of mild infections within the cohort, considering the influence of prophylactic antibiotic use across these time points.

### 2.4 Ethics

From an exclusively healthcare perspective, this dosage adjustment was conducted in a homogeneous and standardized manner at our Unit, following strict criteria established by a committee of experts, which consisted of an internist, a clinical immunologist, and a nurse with extensive experience in treating PID patients. All patients who met the dose adjustment criteria were given the option to either reduce their SCIg dose as previously mentioned or switch to IVIg at the same dose they were receiving subcutaneously. This study was approved by the University and Polytechnic Hospital La Fe Ethical Committee with the code “SUBDOSAGE”. The study was conducted in accordance with the Declaration of Helsinki and adhered to the STROBE guidelines. Anonymity and data confidentiality for all included patients were maintained in compliance with Spanish regulations governing observational studies.

## 3 Results

### 3.1 Study population

Thirty-one patients with PID were included in the study. The mean age was 47.51 years (SD 15.47) with a near equal gender distribution: 16 females (51.61%) and 15 males (48.38%). Among the types of PID observed, 3 patients (9.67%) had IgG subclass deficiency, common variable immunodeficiency (CVID) was identified in 17 patients (54.83%), and 11 patients (35.48%) had other PID with hypogammaglobulinemia. More details are presented in **Table 1**.

**Table 1:** Clinical characteristics of the included patients.

Variable		N (%) – Mean (SD)
Age		47.51 (15.47)
Sex	Female	16 (51.61)
	Male	15 (48.38)
Humoral primary immunodeficiency		
	IgG subclass deficiency	3 (9.67)
	Common variable immunodeficiency	17 (54.83)
	Wiskott-Aldrich syndrome	1
	Chronic mucocutaneous candidiasis	1
	IgA and IgG subclass deficiency	1
	Good syndrome	1
	Di George syndrome	1
	Bloom syndrome	1
	Not well defined hypogammaglobulinemia	5
Comorbidities		
	Cytopenias	6 (19.35)
	Lymphadenopathies	5 (16.12)
	Splenomegaly	1 (3.22)
	Interstitial lung disease	1 (3.22)
	Bronchiectasis	12 (38.70)
	Enteropathy	6 (19.35)
	Neurological affectation	1 (3.22)
	Autoimmune systemic disease	7 (22.58)
	Solid malignancy	2 (6.45)
	Hematological malignancy	3 (9.67)

Regarding their clinical comorbidity, cytopenias were noted in 6 patients (19.35%), lymphadenopathies were present in 5 patients (16.12%), and splenomegaly in 1 patient (3.22%). ILD and neurological affectation were each observed in 1 patient (3.22%). Bronchiectasis was a common comorbidity, affecting 12 patients (38.70%), and 6 patients (19.35%) showed enteropathy. Additionally, 7 patients (22.58%) had autoimmune systemic diseases, and 5 patients of the study had a diagnose of neoplasia: 2 patients (6.45%) had solid malignancies, and 3 patients (9.67%) had hematological malignancies (**Table 1**).

Patients were screened for the use of immunosuppressants before and after the dose adjustment. At baseline, 5 patients were receiving corticosteroids, 1 patient was receiving tacrolimus, and 1 patient was receiving rituximab. At 12 months follow-up, corticosteroid use increased to 6 patients, rituximab and tacrolimus remained unchanged to 1 patient each, and 1 patient started receiving azathioprine.

Additionally, 9 patients (29%) were receiving prophylactic antibiotics at baseline, which non-significantly increased to 10 patients (32.3%) at both the 6-month and 12-month follow-ups (p=0.82). Among these patients, 3 who were not previously on prophylactic antibiotics initiated the regimen, while 2 patients who had been on treatment for the prior 6 months discontinued it (**Table 2**). None of the patients who initiated had presented major bacterial infections.

**Table 2:** Prophylactic antibiotic treatment of our cohort.

	Before dose adjustment (N)	6-month follow-up (N)	12-month follow-up (N)
Azithromycin	4	4	5
Cotrimoxazole	3	3	3
Azithromycin, cotrimoxazole and inhaled colistin	1	1	1
Rifaximin	1	1	0
Ciprofloxacin	0	1	1

N, number of patients of the cohort.

### 3.2 Dose adjustment

The mean dose of SCIg before the dose adjustment was 7.82 g/week (SD 2.29). The mean dose reduction was 2.10 g/week (SD 1.18). Consequently, the new mean dose at 6 months pre-adjustment was 5.72 g/week (SD 1.71). In addition, at 12 months, several dose-adjustments were made: 4 dose escalations (including the patient whose dose was reduced at 6 months) and 1 dose reduction. As so, at 12 months of follow-up, the average SCIg dose was 6.94 g/week (SD 1.91).

Specifically, these dose adjustments were made as follows: one patient with CVID had their dose reduced from 8.3 g/week to 5 g/week, further reduced to 3.75 g/week at 6 months, and then increased to 6.7 g/week at 12 months due to two minor infections. Another CVID patient with renal transplantation had their dose reduced from 8 g/week to 5 g/week, then increased to 8.3 g/week at 12 months due to an increase in BK virus load. A CVID patient with multiple myeloma had her dose reduced from 8 g/week to 7 g/week, then increased back to 8 g/week at 12 months due to a minor infection and to patient preference. A patient with primary non-defined hypogammaglobulinemia and severe asthma had their dose reduced from 7 g/week to 5 g/week, then increased to 6.7 g/week at 12 months due to the development of pneumonia. Finally, one patient had an initial dose reduction from 10 g/week to 6.7 g/week, further reduced to 5 g/week at 12 months, without any subsequent infections.

The data on the type of SCIg and the administration frequency at time 0, as well as the changes in the type of SCIg and administration frequency after the dose adjustment, are detailed in **Table 3**.

**Table 3:** Data on the type of subcutaneous immunoglobulin replacement therapy (SCIg) and administration frequency at time 0, and changes after dose adjustment.

Variable		N	Frequency (%)
Type of SCIg	SCIg 20%	19	61.29
	Hyaluronidase-facilitated SCIg 10%	12	38.71
Frequency of administration	Every 2 weeks	5	16.13
	Every 3 weeks	7	22.58
	Every 4 weeks	5	16.13
	Weekly	14	45.16
Change in the type of SCIg	No	30	96.77
	Yes	1 (changed to Hyaluronidase-facilitated SCIg 10%)	3.23
Change in the frequency of administration	No	25	80.65
	Yes	6	19.35

### 3.3 Presence of infections before and after dose adjustment

There was no significant difference in the number of severe major infections and mild infections before dose adjustment, at 6-months follow-up and at 12 months follow-up (p=0.220 and p=0.107, respectively).

However, regarding major infections, there were none in the 6 months prior to dose adjustment, 2 in the 6 months after dose-adjustment (patient 29 and patient 31), and 1 between 6- and 12-months follow-up (patient 31) in the whole cohort. Patient 31 had previously suffered non-Hodgkin lymphoma and had received autologous stem cell transplantation. All of these were community-acquired pneumonias, and at 12 months, the dose was increased for the patient 31 who had experienced 2 pneumonias within 12 months follow-up. The percentage of patients who suffered severe infections at the three time points was also not statistically significant (p=0.223). There were no cases of other major infections such as meningitis, osteomyelitis, cellulitis, sepsis, UTIs requiring hospitalization, opportunistic infections. Patient 29 received prophylactic antibiotic therapy throughout the entire study period, whereas patient 31 did not receive it at any point during the study period. No significant differences were observed in the cumulative incidence of major infections related to any time points or presence of prophylactic antibiotic therapy.

No patient experienced more than 3 mild infections in any 6-month follow-up period. Additionally, the percentage of patients who suffered mild infections at the 6-month and 12-month follow-ups after dose adjustment was not statistically significant (p=0.109). Regarding these mild infections before the dose adjustment, 3 patients had experienced 1 mild infection, and 1 patient had experienced 2 mild infections. In the first 6 months post-adjustment, 6 patients had 1 mild infection, and 3 patients had 2 mild infections. Between 6- and 12-months post-adjustment, 10 patients had 1 mild infection, and 1 patient had 2 mild infections.

We conducted a generalized linear mixed model to investigate the impact of time on the frequency of mild infections, considering the potential influence of prophylactic antibiotic use over the study period. The results showed no statistically significant differences in the frequency of mild infections across the three time points when accounting for antibiotic use (interaction terms: from 0-6 months, p = 0.9785; from 6-12 months, p = 0.8332). Additionally, the main effect of ATB was not significant (p = 0.9247).

Among these mild infections, one patient had a mild gastrointestinal infection at each time point. For lower respiratory tract infections, there was 1 case before the dose adjustment, compared to 2 cases at 6 months and other 2 cases between 6 and 12 months. There were several cases of mild skin and soft tissue infections: 1 case before the dose adjustment, 3 cases at 6 months, and 2 cases between 6- and 12-months follow-up. Two episodes of mild upper respiratory tract infections were recorded: none in the pre-adjustment period, with 1 case each at 6 months and between 6 and 12 months. There was only 1 case of a mild urinary tract infection, which occurred in a patient during the 6 months post-adjustment period. No cases of mild parasitic infections were observed (**Table 4**).

**Table 4:** Number of infections recorded before dose adjustment, and at 6 and 12 months follow-up.

	Pre-adjustment			0-6 months post-adjustment			6-12 months post-adjustment		
Number of infections	0	1	2	0	1	2	0	1	2
Type of infection									
**Major infection**	31 (100%)	0 (0%)	0 (0%)	29 (93.55%)	2 (6.45%)	0 (0%)	30 (96.77%)	1 (3.23%)	0 (0%)
Pneumonia	31 (100%)	0 (0%)	0 (0%)	28 (90.32%)	2 (6.45%)	0 (0%)	30 (96.77%)	1 (3.23%)	0 (0%)
Meningitis	31 (100%)	0 (0%)	0 (0%)	31 (100%)	0 (0%)	0 (0%)	31 (100%)	0 (0%)	0 (0%)
Osteomyelitis	31 (100%)	0 (0%)	0 (0%)	31 (100%)	0 (0%)	0 (0%)	31 (100%)	0 (0%)	0 (0%)
Severe abdominal infection	31 (100%)	0 (0%)	0 (0%)	31 (100%)	0 (0%)	0 (0%)	31 (100%)	0 (0%)	0 (0%)
Severe skin and soft tissue infection	31 (100%)	0 (0%)	0 (0%)	31 (100%)	0 (0%)	0 (0%)	31 (100%)	0 (0%)	0 (0%)
Sepsis	31 (100%)	0 (0%)	0 (0%)	31 (100%)	0 (0%)	0 (0%)	31 (100%)	0 (0%)	0 (0%)
Severe urinary tract infection	31 (100%)	0 (0%)	0 (0%)	31 (100%)	0 (0%)	0 (0%)	31 (100%)	0 (0%)	0 (0%)
Opportunistic infection	31 (100%)	0 (0%)	0 (0%)	31 (100%)	0 (0%)	0 (0%)	31 (100%)	0 (0%)	0 (0%)
**Non-severe infection**	27 (87.1%)	3 (9.68%)	1 (3.23%)	22 (70.97%)	6 (19.35%)	3 (9.68%)	20 (64.52%)	10 (32.26%)	1 (3.23%)
Mild upper respiratory tract infection	29 (93.55%)	2 (6.45%)	0 (0%)	26 (83.87%)	4 (12.9%)	1 (3.23%)	25 (80.65%)	5 (16.13%)	1 (3.23%)
Mild lower respiratory tract infection	30 (96.77%)	1 (3.23%)	0 (0%)	29 (93.55%)	2 (6.45%)	0 (0%)	29 (93.55%)	2 (6.45%)	0 (0%)
Mild gastrointestinal infection	30 (96.77%)	1 (3.23%)	0 (0%)	30 (96.77%)	1 (3.23%)	0 (0%)	30 (96.77%)	1 (3.23%)	0 (0%)
Mild urinary tract infection	31 (100%)	0 (0%)	0 (0%)	30 (96.77%)	1 (3.23%)	0 (0%)	31 (100%)	0 (0%)	0 (0%)
Parasitosis	31 (100%)	0 (0%)	0 (0%)	31 (100%)	0 (0%)	0 (0%)	31 (100%)	0 (0%)	0 (0%)
Mild skin and soft tissue infection	30 (96.77%)	1 (3.23%)	0 (0%)	28 (90.32%)	3 (9.68%)	0 (0%)	29 (93.55%)	2 (6.45%)	0 (0%)
**Recurrent infection (more than 3 in 6 months)**	0 (0%)	0 (0%)	0 (0%)	0 (0%)	0 (0%)	0 (0%)	0 (0%)	0 (0%)	0 (0%)

Bolded terms represent overarching categories that group related infection types for classification purposes.

### 3.4 Analysis of immunological parameters

**Figure 2:**
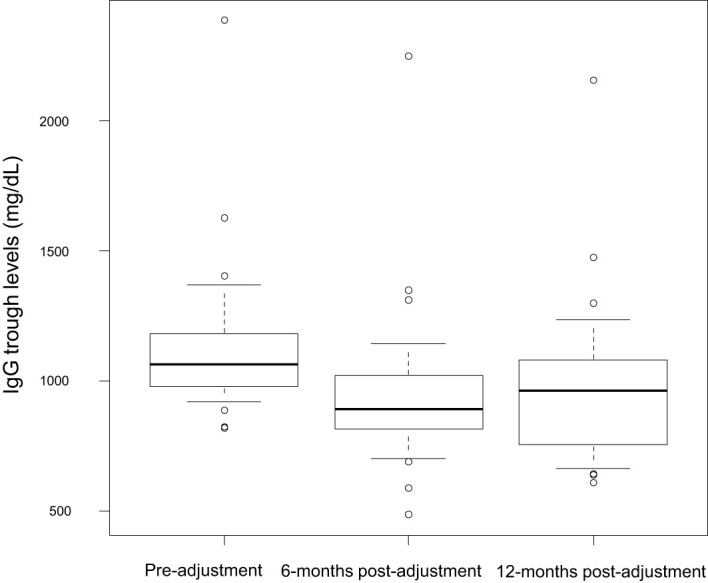
IgG trough levels at baseline, 6- and 12-months follow up.

On the one hand, after the dose adjustment, a statistically but not clinically significant difference was observed in the trough levels of IgG across the different time points in follow-up: 1,138.48 (SD 294.25) at baseline, 947.35 (SD 306.69) 6-months post-adjustment, and 983.28 (SD 310.51) at 12-months post-adjustment (p = 0.002), with the lowest IgG trough levels recorded at 6 months (**Figure 2**). The mean percentage of reduction of the IgG trough levels at 0-6 months was 16.77% (SD 13.88%) and at 6-12 months was 13.76% (SD 16.78%). This difference was not clinically relevant in the number of severe and mild infections. However, it is important to remark that patient 31 who suffered two major infections had a markedly higher reduction of 53.71% of the baseline IgG trough levels, even under normal levels (484 mg/dL) and required subsequent increase of IgRT dosing. Patient 29 IgG trough levels did not fall under normal values nor were reduced by more than 1 standard deviation of the mean percentual reduction of the cohort.

On the other hand, mean total leukocyte counts were 6288.71 (SD 1752.84), 7037.24 (SD 1887.27), and 6662.50 (SD 2171.55) cells/μL at baseline, 6 months, and 12 months, respectively. Baseline mean CD4, CD8, and NK cell counts were 846.72 (SD 490.69), 510.03 (SD 292.55), and 245.10 (SD 201.99) cells/μL, respectively. In patients 29 and 31, no subsequent lymphopenia were observed.

### 3.5 Pharmacoeconomic analysis

In our study, a standardized dose reduction of Igs was implemented for a total of 31 patients with humoral PID, maintained for at least one year. The average dose reduction was 2.09 g/week (SD 1.17) resulting in an average annual savings of 100.3 g per patient. For the entire cohort of 31 patients, this equated to an annual savings of approximately 3109.3 g. From a pharmacoeconomic perspective, using the reference price of 55.5 euros/g as established by the Spanish Ministry of Health, this translates to an average savings of 5,550 euros per patient per year, and a total annual savings of 172,050 euros for our cohort (19).

## 4 Discussion

The main findings of this study can be summarized as follows: (i) The implementation of a careful, clinically tailored and standardized dose reduction SCIg therapy in patients with humoral PIDs was successfully maintained over an extended period without an increase in severe or mild infections, and (ii) individualized SCIg dose reduction could be a safe and cost-effective approach to managing PIDs, especially in contexts where plasma-derived products are scarce. These results highlight the potential of personalized dosing strategies to optimize patient care and resource utilization effectively.

IgRT is essential for managing PIDs with hypogammaglobulinemia, as it significantly reduces the frequency and severity of infections, mitigates organ damage, and decreases mortality (7, 11). It is well established that higher doses of immunoglobulins correlate with increased serum IgG levels and reduced infection rates, regardless of the administration route (11, 16). However, there is no universally defined protective IgG level. Most practice guidelines recommend maintaining trough IgG levels between 600 and 800 mg/dL, achieved by administering 400 mg/kg every 3 to 4 weeks (6, 14, 15). This recommendation is largely based on expert opinions and systematic reviews of limited data. Consequently, dose optimization is usually guided by the so-called “minimum biological trough levels,” which represent the specific IgG concentration required to prevent infections in each patient (17, 18). The variability in individual IgG thresholds implies that some patients might be under-treated, increasing their risk of infections, while others could be over-treated, leading to inefficient use of IgRT.

The British Society for Immunology and the UK Primary Immunodeficiency Network (UKPIN) recommend maintaining initial trough IgG levels above 800 mg/dL for PID patients, with potential optimization to 1000 mg/dL in cases of X-linked agammaglobulinemia, established organ damage, or persistent infections (20). In fact, recommendation 28 suggests considering immunoglobulin dose reduction for stable, infection-free patients with primary or secondary immunodeficiency. However, a standardized protocol for patient selection and specific dose reduction strategies is not provided.

In this regard, Elhaj et al. reduced IgRT doses for 61 patients, 48 of whom had humoral PID, during the COVID-19 Ig shortage. This reduction was applied to patients with IgG trough levels above 800 mg/dL, provided their condition was clinically stable (21). In this study, the dose reduction group had significantly higher baseline trough levels compared to controls (1050 mg/dL vs. 820 mg/dL). After dose reduction, trough IgG levels in the reduction group decreased to 860 mg/dL, comparable to the control group (890 mg/dL). In our study, baseline trough levels were higher (1130 mg/dL), decreasing to 940 mg/dL at six months and 980 mg/dL at twelve months. The reduction magnitude was similar to the British study (-190 mg/dL vs. -150 mg/dL). However, in their study, breakthrough infections and the need for antibiotics occurred 1.55 times more frequently in the reduced dose group compared to controls, with a significant increase in antibiotic use post-IgRT dose reduction. In contrast, our study found no significant differences in infection rates before and after dose reduction, regardless of prophylactic antibiotic use.

This discrepancy could be attributed to our patients having higher absolute trough IgG levels and the strict inclusion of humoral PID patients. Contrarily, the study by Elhaj et al. included secondary immunodeficiency patients, who may have additional immunosuppressive factors necessitating prophylactic antibiotic use for infection prevention. Additionally, dose reduction was not individualized based on clinical factors (e.g., organ damage, comorbidities), but rather applied uniformly. This approach may have led to inappropriate patient selection and inadequate dose reductions, either by including patients who were not properly adjusted or by reducing doses excessively in particularly vulnerable patients.

IgRT has complex production processes and relies on plasma donations, making it a scarce and valuable resource. Spain, in particular, faces vulnerability with low plasma self-sufficiency (34%) and heavy reliance on importation (22). Recent measures, such as use optimization plans and plasma donation campaigns, have shown limited effectiveness. In our study, we carefully selected candidates for Ig dose reduction, including only humoral PID patients with IgG trough levels ≥700 mg/dL and no significant infections in the past six months. For patients with GLILD, inflammatory enteropathy, recent immunosuppressant use, or bronchiectasis, trough levels ≥900 mg/dL were required, with decisions made by a clinical expert committee. Our results, that highlight one patient who experienced two non-severe pneumonias and who had a history of non-Hodgkin lymphoma and had undergone autologous stem cell transplantation make us suggest that these comorbidities should also be considered when evaluating potential IgG dose reductions.

This work demonstrates that standardized Ig dose reduction in selected well-controlled humoral PID patients could be a reasonable strategy to conserve Ig resources without compromising patient safety. This approach could significantly improve resource management and offer a potential solution to the Ig supply challenge. The total annual savings in our experience amounted to 172,050 euros for the Spanish National Health System, substantially exceeding the estimated direct costs of inpatient and outpatient breakthrough infections such as pneumonia, which are estimated at 3,955 euros for hospital admission and 511 euros for outpatient management in our setting (23).

However, this study is not exempt from limitations. Firstly, this is a retrospective observational study based on data analysis following the implementation of a contingency plan, designed from a primarily clinical perspective during the exceptional context of the 2021 Ig shortage crisis among COVID-19 pandemic. Secondly, the study included only patients with humoral PIDs, making it difficult to extrapolate the results to patients with SID, as these conditions involve additional iatrogenic non-humoral factors that could significantly influence infection rates. Additionally, the sample size was small, and the study lacks a control group, as all patients offered dose reduction accepted it, limiting the extrapolation of our findings to established clinical practices and recommendations. In fact, the analysis included only patients receiving SCIg, making it difficult to extrapolate the results to patients receiving IVIg, showing different pharmacokinetic profiles that could lead to different outcomes in IgG trough levels and infection rates following dose reduction. Furthermore, the analysis included only patients receiving SCIg, making it challenging to generalize the results to those receiving IVIg, which has distinct pharmacokinetic profiles that could lead to different outcomes in IgG trough levels and infection rates following dose reduction. Additionally, this study was conducted in 2021, a period when face-mask use was widely recommended to immunosuppressed patients by their physicians in public settings. This precaution may have contributed to the lack of a significant increase in infection rates observed in our study. Lastly, while our findings provide valuable preliminary insights, long-term prospective studies with larger and more diverse cohorts, including patients receiving IVIg and those with SIDs, are crucial to validate these results and enable their translation into clinical practice. Such studies are essential to identify patients who may benefit from tailored dose adjustments, addressing the current lack of evidence on this topic. Rigorous prospective studies with well-defined inclusion criteria will be indispensable in assessing the safety of this approach in larger populations. Moreover, these studies will offer critical insights into pharmacoeconomic outcomes, optimizing resource allocation and enhancing patient care.

## 5 Conclusion

This study suggests that SCIg dose optimization in patients with humoral PIDs requiring IgRT and a low infectious burden may be feasible without increasing severe or recurrent infections, provided that specific clinical criteria are met, including careful patient selection and a standardized, proportionate dose reduction based on IgG trough levels, alongside close monitoring and follow-up. Candidates for dose reduction should have maintained stable IgG trough levels of at least 700 mg/dL, or 900 mg/dL for those with GLILD, inflammatory enteropathy, recent use of immunosuppressants -or history of malignancy or stem cell transplantation-, or bronchiectasis, and should not have experienced severe or recurrent infections in the past six months. Once selected, the dose reduction should be standardized, reducing 60 mg/kg/month (15 mg/kg/week) for every 150 mg/dL above the target trough levels of 700 mg/dL or 900 mg/dL.

Given the limitations of this study, our findings should be interpreted with caution. Nonetheless, the data provided could be highly valuable, highlighting the possibility that many of our patients with humoral PIDs might be receiving excessively high doses. This practice could result in suboptimal efficiency and threaten the sustainability of future treatments. Furthermore, implementing prospective studies to evaluate these strategies is challenging but necessary. Additional research with larger patient cohorts is required to confirm the utility of this reduction approach, extending beyond humoral PID patients to include those with secondary immunodeficiencies, while also assessing different formulations and routes of administration.

## Data Availability

The original contributions presented in the study are included in the article/supplementary material. Further inquiries can be directed to the corresponding author/s.
